# *Mycobacterium tuberculosis* curli pili facilitates pathogenicity by modulating central carbon metabolism

**DOI:** 10.1007/s11306-025-02320-5

**Published:** 2025-08-12

**Authors:** Tarien J. Naidoo, Shinese Ashokcoomar, Barry Truebody, Jared S. Mackenzie, Adrie J. C. Steyn, Manormoney Pillay

**Affiliations:** 1 Medical Microbiology, School of Laboratory Medicine and Medical Sciences, College of Health Sciences, University of KwaZulu-Natal, 1st Floor Doris Duke Medical Research Institute, Congella, Private Bag 7, Durban, 4013 South Africa; 2 Africa Health Research Institute, Durban, South Africa; 3 Department of Microbiology, Center for AIDS Research and Free Radical Biology, University of Alabama at Birmingham, Birmingham, USA

**Keywords:** Mycobaterium tuberculossis, MTP, Bioenergetics, LS-MS/MS, Central carbon metabolism

## Abstract

**Introduction:**

Strategies specifically targeting the initial host–pathogen interactions, hold great promise in the identification of accurate biomarkers for tuberculosis (TB) prevention interventions. *Mycobacterium tuberculosis* (*Mtb*) curli pili (MTP) (encoded by *mtp/Rv3312A*), a surface adhesin utilised by the pathogen to interact with host receptor cells, has been reported as a suitable target for TB diagnostic and therapeutic strategies. Previous “omics” studies highlighted the role MTP potentially plays in *Mtb* central carbon metabolism (CCM). However, its precise contribution to metabolism remains unknown.

**Objectives:**

This study aimed to examine the role of MTP in the bioenergetic metabolism of *Mtb*, using bedaquiline (BDQ) to inhibit ATP production through oxidative phosphorylation (OXPHOS), extracellular flux analysis, *Mtb* wildtype (WT), ∆*mtp* deletion mutant, and *mtp-*complemented strains. The role of MTP in regulation of CCM was assessed using ^13^C_6_-metabolic flux analysis.

**Results:**

MTP was associated with increased bacterial respiration and decreased carbon catabolism via glycolysis in response to the inhibition of ATP synthase by BDQ. The dependence of *Mtb* Δ*mtp* on OXPHOS for energy production was demonstrated to be greater than the WT and *mtp-*complemented strains. In addition, metabolic flux profiles revealed that in the Δ*mtp* mutant, CCM was dysregulated by decreasing flux through glycolysis, tricarboxylic acid cycle, glyoxylate and dicarboxylate metabolism, and the pentose phosphate pathway in comparison to the WT.

**Conclusion:**

These novel findings show that MTP is associated with the regulation of bioenergetics and metabolism pathways and substantiate MTP as a potential biomarker for TB diagnostics/therapeutics, and a novel target for vaccine/drug development.

**Supplementary Information:**

The online version contains supplementary material available at 10.1007/s11306-025-02320-5.

## Introduction

The eradication and control of tuberculosis (TB), caused by *Mycobacterium tuberculosis* (*Mtb*), is greatly threatened by the emergence and continual spread of drug resistance (Mitnick et al., 2008; WHO, 2023). Therefore, there is an urgent need to identify novel biomarkers for the development of improved TB diagnostics, vaccines, and therapeutics. Targeting the initial host–pathogen interaction holds great promise in the identification of novel biomarkers (Krachler & Orth, 2013). Functional genomics (Govender et al., 2014, 2018; Ramsugit et al., 2013, 2016), transcriptomics (Moodley, 2018; Naidoo et al., 2014, 2025), and metabolomics (Ashokcoomar et al., 2020, 2021; Reedoy et al., 2020) have identified the adhesin *Mtb* curli pili (MTP) as suitable target for the development of TB diagnostics, therapeutics, and intervention strategies. The MTP adhesin exhibits great affinity to laminin which allows for the adherence to pulmonary epithelial cells (Alteri, 2005). The MTP adhesin is encoded by the conserved *mtp* gene and is found exclusively in members of the *Mtb* Complex strains (Naidoo et al., 2014). Moreover, MTP is known to play a role in biofilm production (Ramsugit et al., 2013), adhesion to and invasion of both THP-1 macrophages (Ramsugit & Pillay, 2014) and A549 pulmonary epithelial cells (Ramsugit et al., 2016). This adhesin additionally reduces the induction of cytokine/chemokine as a survival strategy in epithelial cells (Ramsugit et al., 2016).

Further to being classified as an adhesin, transcriptomic (Naidoo et al., 2025) and metabolomic (Ashokcoomar et al., 2020) studies have reported significant evidence revealing the potential role MTP plays in modulating metabolism. The absence of MTP perturbs the electron transport chain (ETC) potentially altering the proton gradient which subsequently leads to metabolic changes (Naidoo, 2021). Classified as an obligate aerobe, *Mtb* requires the use of its branched ETC for energy production through oxidative phosphorylation (OXPHOS) (Cook et al., 2014). Glycolysis and OXPHOS are the two central metabolic pathways responsible for ATP generation aiding in the growth and pathogenicity of *Mtb* (Cook et al., 2014). In the present study, it was hypothesised that MTP modulates *Mtb’s* CCM by enhancing glycolysis and the tricarboxylic acid (TCA) cycle aiding its growth and pathogenicity. To test this hypothesis, extracellular flux analysis using bedaquiline (BDQ) to inhibit ATP production, and ^13^C-metabolic flux analysis were performed.

Numerous studies demonstrated that the diarylquinolone, BDQ, inhibits the production of ATP through OXPHOS (Andries et al., 2005; Koul et al., 2008; Lamprecht et al., 2016; Mackenzie et al., 2020; Watanabe et al., 2011). BDQ inhibits OXPHOS by targeting the ETC complex V, ATP synthase, thereby starving the pathogen of ATP (Koul et al., 2008; Matteelli et al., 2010). It is postulated that BDQ causes back pressure on the ETC resulting in the inhibition of the proton motive force and decreased metabolic flux (Berney et al., 2014; Cook et al., 2014; Koul et al., 2008). Therefore, using BDQ to inhibit OXPHOS, this study used extracellular flux analysis to measure oxygen consumption rate (OCR) and extracellular acidification rate (ECAR) in real-time in an effort to determine the effect of MTP on the bioenergetics of *Mtb*. Moreover, ^13^C_6_-glucose tracing followed by ^13^C-metabolic flux analysis was performed to determine the role MTP plays in regulation of CCM, specifically glycolysis, the TCA cycle, pentose phosphate pathway (PPP), glyoxylate shunt, and amino acids.

## Materials and methods

### Ethics approval

The study was approved by the University of KwaZulu-Natal Biomedical Research Ethics Committee (BREC/00004043/2022). The bacterial culturing and experimental procedures were conducted in a Biosafety level 2 + (BSL2 +) laboratory at Medical Microbiology, University of KwaZulu-Natal, Durban, South Africa, and a BSL3 + laboratory at Africa Health Research Institute (AHRI), Nelson R. Mandela School of Medicine, Durban, South Africa.

### Bacterial strains and growth conditions

The three bacterial strains used in the study included the *Mtb* wildtype V9124 (WT), a clinical isolate of the F15/LAM4/KZN family previously isolated in Medical Microbiology, University of KwaZulu-Natal, from Tugela Ferry (KwaZulu-Natal, South Africa) (Gandhi et al., 2006), the *mtp* gene knockout mutant (∆*mtp*) and the corresponding *mtp-*complemented strain. Briefly, the Δ*mtp* (Ramsugit et al., 2013) strain was constructed via specialised transduction through replacement of *mtp* with an allelic exchange substrate (AES) containing the hygromycin-resistance (HygR)-sacB cassette (Bardarov et al., 2002). The *mtp*-complemented (Ramsugit et al., 2013) strain was constructed via electrotransformation by insertion of the pMV261 plasmid containing the *mtp* gene (Bardarov et al., 2002). The strains were cultured in Middlebrook 7H9 or 7H11 medium (Difco, Becton–Dickinson, Franklin Lake, New Jersey, USA) supplemented with 10% (v/v) oleic albumin dextrose catalase (OADC) (Difco, Becton–Dickinson, Franklin Lake, New Jersey, USA), 0.5% (v/v) glycerol (Sigma-Aldrich, Missouri, USA), and 0.05% (v/v) tween-80 (Sigma-Aldrich, Missouri, USA). The Δ*mtp* and *mtp*-complemented strains were grown in the presence of the antibiotics hygromycin (75 μg mL^−1^) and kanamycin (30 μg mL^−1^), respectively. The bacterial cultures were incubated at 37 °C in a shaking incubator (I-26 Shaking Incubator, New Brunswick Scientific, Canada) at 1 × g until an optical density (OD)_600 nm_ of 1.0, equivalent to approximately 1 × 10^8^ colony forming units (CFU)/mL (Larsen et al., 2007). The Lightwave II Spectrophotometer (Biochrom Ltd, Cambridge, United Kingdom) was used to determine the OD. The bacterial strains were then confirmed by gene specific polymerase chain reaction (PCR) using the genomic deoxyribonucleic acid (DNA) extracted via InstaGene Matrix (Bio-Rad Laboratories, Hercules, California, USA.

### *Mtb* oxygen consumption rate (OCR) and extracellular acidification rate (ECAR) measurements

The *Mtb* strains were cultured in supplemented Middlebrook 7H9 media (Difco, Becton–Dickinson, Franklin Lake, New Jersey, USA), to an early log phase with an OD_600nm_ of ~ 0.6 prior to experiments. The bottom of the extracellular flux (XF) cell culture microplate (Agilent) was coated with Cell-Tak (Agilent) to allow adherence of the bacilli to the microplate. The Cell-Tak solution has no effect on the basal respiration of *Mtb* (Lamprecht et al., 2016). The *Mtb* bacilli were adhered to the XF microplate at 4 × 10^6^ bacilli per well. The experimental assays in the Extracellular Flux Analyzer 96 (Agilent) were carried out in unbuffered 7H9 media (pH 7.4). The basal OCR and ECAR were measured at baseline level for 21 min, prior to the addition of the ETC targeting drugs through the drug ports of the sensor cartridge. The ETC targeting BDQ, a diarylquinolone, inhibits the ATP Synthase (Complex V of the OXPOS pathway) by binding to the subunit c^11^ (Preiss et al., 2015), thereby starving the bacteria of ATP (Koul et al., 2008; Matteelli et al., 2010). A final concentration of 5.4 µM of BDQ was injected through the drug ports and the assay was concluded with the addition of the uncoupler carbonyl cyanide m-chlorophenyl hydrazone (CCCP) at a final concentration of 4 µM. The uncoupler depolarizes the cell membrane and was optimized to this concentration to increase maximal respiration and carbon catabolism that is sustainable by the bacteria. The OCR and ECAR were measured for an additional 35 min.

### *Mtb* metabolite extraction

The *Mtb* cultures were grown to an OD_600nm_ of ~ 0.6, as described above. The bacterial cultures were pelleted and washed twice using phosphate buffered saline (PBS) solution (Thermo Fisher Scientifc, Waltham, MA, USA). Thereafter, the pellets were suspended in 4.5 mL of 7H9 Middlebrook media (Difco, Becton–Dickinson, Franklin Lake, New Jersey, USA) containing the ^13^C_6_-glucose (LC Scientific, Nairobi, Kenya) tracer carbon source to a final concentration of 0.2%. The cultures were incubated for ~ 16 h at 37 °C in a shaking incubator (I-26 Shaking Incubator, New Brunswick Scientific, Canada) at 1 × g, prior to sampling. The cultures were centrifuged at 3000 × g for 10 min and the pellets were snap frozen and kept on dry ice. The frozen pellets were suspended in a volume of 1.8 mL of extraction buffer containing 2:2:1 methanol:acetronitrile:water, and the internal standards of D4-Alanine and D4-Succinate (Sigma-Aldrich, Missouri, USA). The bacilli were lysed via five rounds of bead beating at 7000 rpm for 60 s followed by 4 min of cooling on dry ice. The cells were then centrifuged for 10 min at 20,000 × g. The culture supernatants were sterilized using 0.2-μm membrane-spin filters and centrifugation at 3000 × g for 30 min. The protein concentrations were determined using the Micro BCA™ Protein Assay Kit (Thermo Scientific, Waltham, MA, USA). The cell filtrates were then evaporated at 42 °C for ~ 16 h. The pellets were resuspended in 100 µL distilled water for organic acid analysis by LC–MS/MS. A volume of 30 µL of suspended cell lysates was diluted with 70 µL acetonitrile for amino acid analysis by LC–MS/MS. Protein concentrations of the samples, measured using the Micro BCA™ Protein Assay Kit (Thermo Scientific, Waltham, Massachusetts, USA), were used to normalise the metabolomics data.

### LC–MS/MS

Labelled organic acids and amino acids were analysed using LC–MS/MS. The organic acid samples from the *Mtb* extracts were separated on a Biorad Aminex HPX-87 column (300 × 7.8 mm) (Sigma-Aldrich, Missouri, USA). This was achieved using a 0.1% aqueous formic acid isocratic mobile phase programme (Mackenzie et al., 2020) with an injection volume of 20 µL and a flow rate of 400 µL/minute. The amino acid samples were separated on a Waters BEH Amide column (2.1 × 100 mm, 1.7 µm) (Sigma-Aldrich, Missouri, USA). This was achieved using a gradient-elution programme with an injection volume of 1 µL and a flow rate of 200 µL/minute and using two mobile phases. Mobile-phase A included the aqueous 0.1% formic acid and mobile-phase B included the acetonitrile and 0.1% formic acid. The gradient programme included: 0–0.2 min, 99% of mobile-phase B; 0.2–24 min, followed by a curvilinear decrease from 99–30% of mobile-phase B; 24–25 min, 30% of mobile-phase B; 25.1–35 min, 99% of mobile-phase B. The Aminex and the BEH Amide columns were connected to a Dionex Ultimate 3000 UPLC (Thermo Scientific, Waltham, Massachusetts, USA). The total ion chromatograms (50–750 m/z scan range), which included the negative ions (organic acids) and positive ions (amino acids), were collected using a Thermo Scientific Q Exactive mass spectrometer (Thermo Scientific, Waltham, Massachusetts, USA). Skyline version 3.7 software (MacCoss Lab, University of Washington, Seattle, WA, USA) was used to visualise and analyse the total ion chromatograms of all metabolites and the respective isotopologues. The chromatographic data was then exported and analysed using Windows Excel 2016. Data representation and statistical analysis were performed using GraphPad Prism Version 8 software (GraphPad Software, La Jolla, CA, USA) and MetaboAnalyst Version 4 (Chong et al., 2018).

### Statistical analysis

All experiments were performed independently a minimum of two times with five to eight biological replicates. The data presented is representative of one independent experiment. Statistical analysis was performed using GraphPad Prism Version 8 software (GraphPad Software, La Jolla, CA, USA) and a one-way analysis of variance (ANOVA) with Tukey’s multiple comparison (Supplementary information (SI) Tables S1-S7). False discovery rate (FDR) adjusted *p* < 0.05 was considered statistically significant.

## Results

### The MTP adhesin is associated with an increased bacterial respiration and decreased carbon catabolism in *Mtb*

**Fig. 1:**
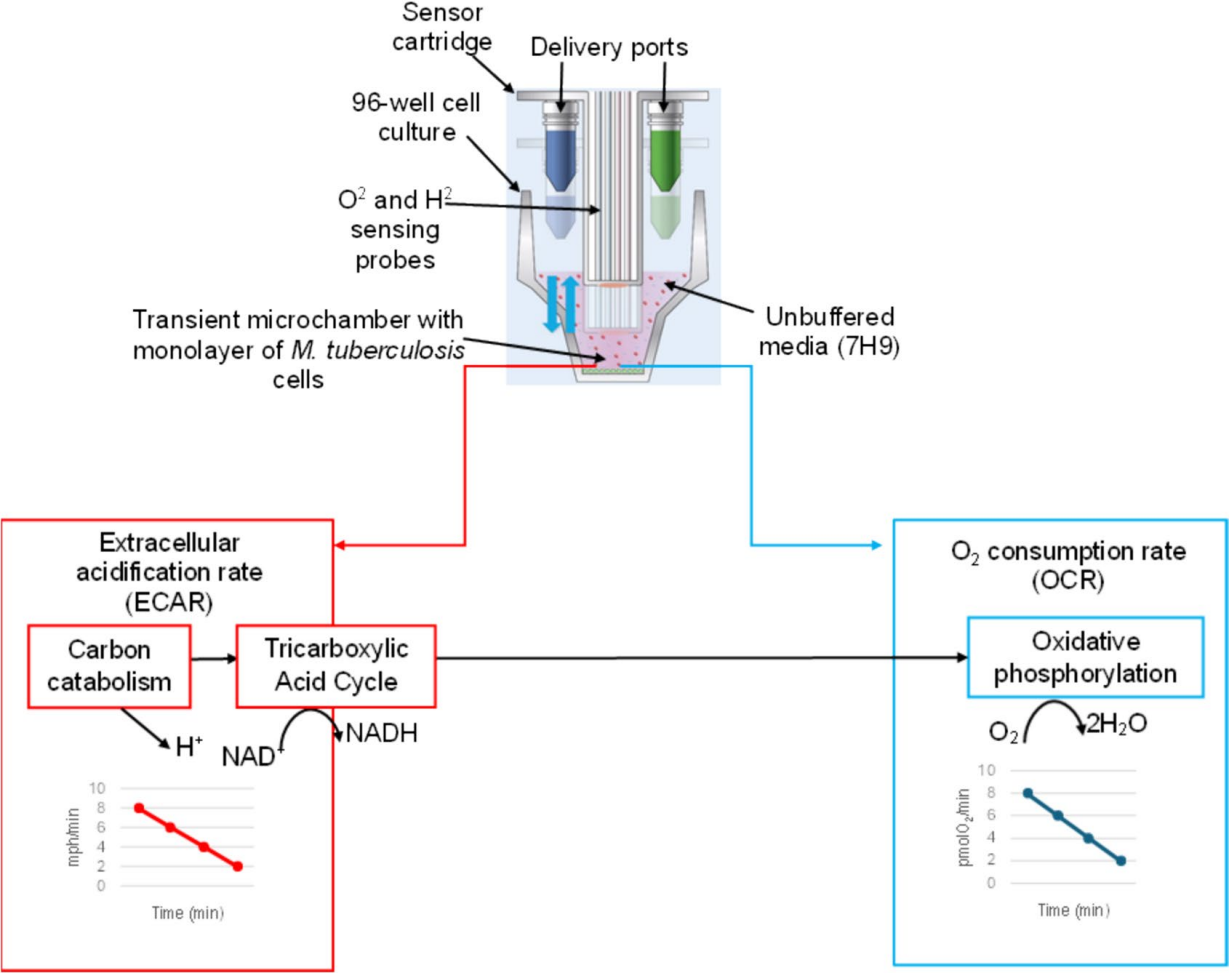
Illustration of a probe of the Agilent Seahorse XF96 Analyzer cartridge and a well of the 96-well culture microplate. During the assay, the compounds are delivered into the microplate via the drug ports. When the probe is lowered, a transient microchamber forms above the monolayer of cells. The probe measures the pH and dissolved O_2_. The instrument software calculates the oxygen consumption rate (OCR) and extracellular acidification rate (ECAR) from these measurements. The OCR is measured as an indication of OXPHOS and ECAR is measured as an indication of the H^+^ production from carbon catabolism

**Fig. 2:**
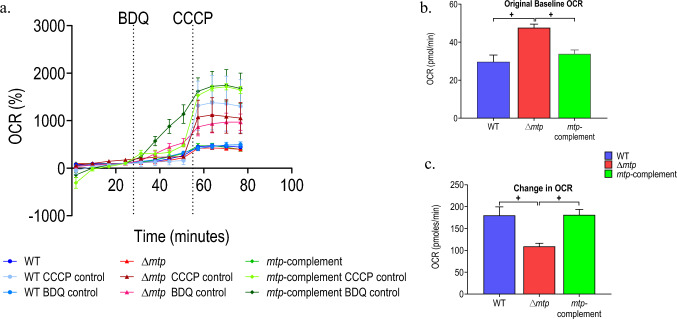
**a** Baselined OCR profiles of *Mtb* wildtype V9124 (WT), ∆*mtp* deletion mutant and *mtp-*complemented strains after the addition of ETC-targeting drug BDQ (10 X MIC, 5.4 µM) followed by the addition of the uncoupler carbonyl cyanide m-chlorophenyl hydrazone (CCCP) (4 µM), after normalization with CFUs. **b** Original baseline, prior to BDQ addition, OCR measurements, and **c** change in OCR of *Mtb* strains after the addition of BDQ (10 X MIC, 5.4 µM). CCCP depolarises the cell membrane to increase respiration and carbon catabolism to the maximum sustainable by the bacterium. The drugs/compounds were delivered through drug ports into the microplate. The probe measured the dissolved oxygen and pH levels that were used in the OCR calculations. The drug additions are indicated by the vertical dotted lines. The profiles and the OCR measurements are representative of one independent assay per 4 million cells per well. The change of OCR in response to BDQ was calculated using measurement 8, just before the second injection (maximal response) and measurement 4, just before the first injection (baseline). Tukey’s multiple-comparisons test with adjusted *p*-values * *p* < 0.05, + *p* ≤ 0.01, # *p* ≤ 0.0001

**Fig. 3:**
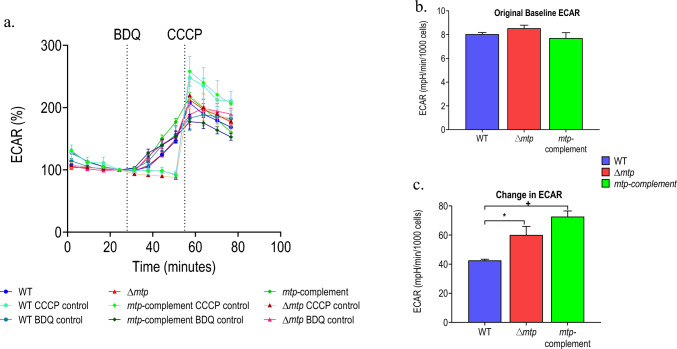
**a** Baselined ECAR profiles of *Mtb* wildtype V9124 (WT), ∆*mtp* deletion mutant, and *mtp-*complemented strains after the addition of ETC-targeting drug BDQ (10 X MIC, 5.4 µM) followed by the addition of the uncoupler carbonyl cyanide m-chlorophenyl hydrazone (CCCP) (4 µM), after normalization with CFUs. **b** Original ECAR, prior to BDQ addition, measurements, and **c** change in ECAR of *Mtb* strains after the addition of BDQ (10 X MIC, 5.4 µM). The drugs/compounds were delivered through drug ports into the microplate. The probe measured the dissolved oxygen and pH levels for the calculation of the ECAR. The drug additions are indicated by the vertical dotted lines. The profiles and the ECAR measurements are representative of one independent assay per 4 million cells per well. The change of ECAR in response to BDQ was calculated using measurement 8 (maximal response) and measurement 4 (baseline). Tukey’s multiple comparisons test with adjusted *p*-values relative to the WT: * *p* < 0.05, + *p* ≤ 0.01, # *p* ≤ 0.0001

To determine the effect of MTP on the bioenergetics of *Mtb,* the XF96 Extracellular Flux Analyzer was used to measure the OCR and ECAR in real-time as markers of OXPHOS and carbon catabolism (Lamprecht et al., 2016; Saini et al., 2016) (Figs. 1, 2, 3). The overall bioenergetic analysis revealed a significant difference in the OCR and ECAR measurements of the ∆*mtp* and WT in response to BDQ and carbonyl cyanide m-chlorophenyl hydrazone (CCCP) (final concentration of 4 µM) (Fig. 2a and 3a). Although the ∆*mtp* displayed the highest basal OCR, its reaction to BDQ was significantly different to the WT (Fig. 2a and 3a). The addition of the uncoupler CCCP to stimulate respiration resulted in a substantial increase in OCR to the maximum sustainable by the pathogen (Fig. 2a and 3a).

The basal OCR measurements (prior to baselining, Fig. 2b) were significantly higher for the ∆*mtp* strain compared to the WT (*p* = 0.0005) and *mtp*-complement (*p* = 0.0035) strains. The basal ECAR measurements (prior to baselining, Fig. 3b) showed no significant difference amongst the strains. The baselined OCR and ECAR measurements (Figs. 2c and 3c) respectively, are reported as a percentage of basal levels.

The baseline OCR profile (Fig. 2b) and change in OCR (Fig. 2c) illustrated the decreased response of ∆*mtp* to the ETC targeting drug, suggesting that in response to inhibition of ATP synthase, the mutant demonstrates a significant decrease in respiration in comparison to the WT (*p* = 0.0058) and *mtp*-complement (*p* = 0.0036). The baseline ECAR profile (Fig. 3b) and the change in ECAR (Fig. 3c) depicted an increased response in the ∆*mtp* compared to the WT. Although the strains displayed no significant differences in the basal measurement, the addition of BDQ and subsequent CCCP addition significantly altered their responses. The ∆*mtp* (*p* = 0.0297) demonstrated a significantly higher change in ECAR compared to the WT, indicating a significant increase in carbon catabolism in response to the inhibition of OXPHOS.

### The MTP adhesin is associated with enhanced glycolytic flux in *Mtb*

**Fig. 4:**
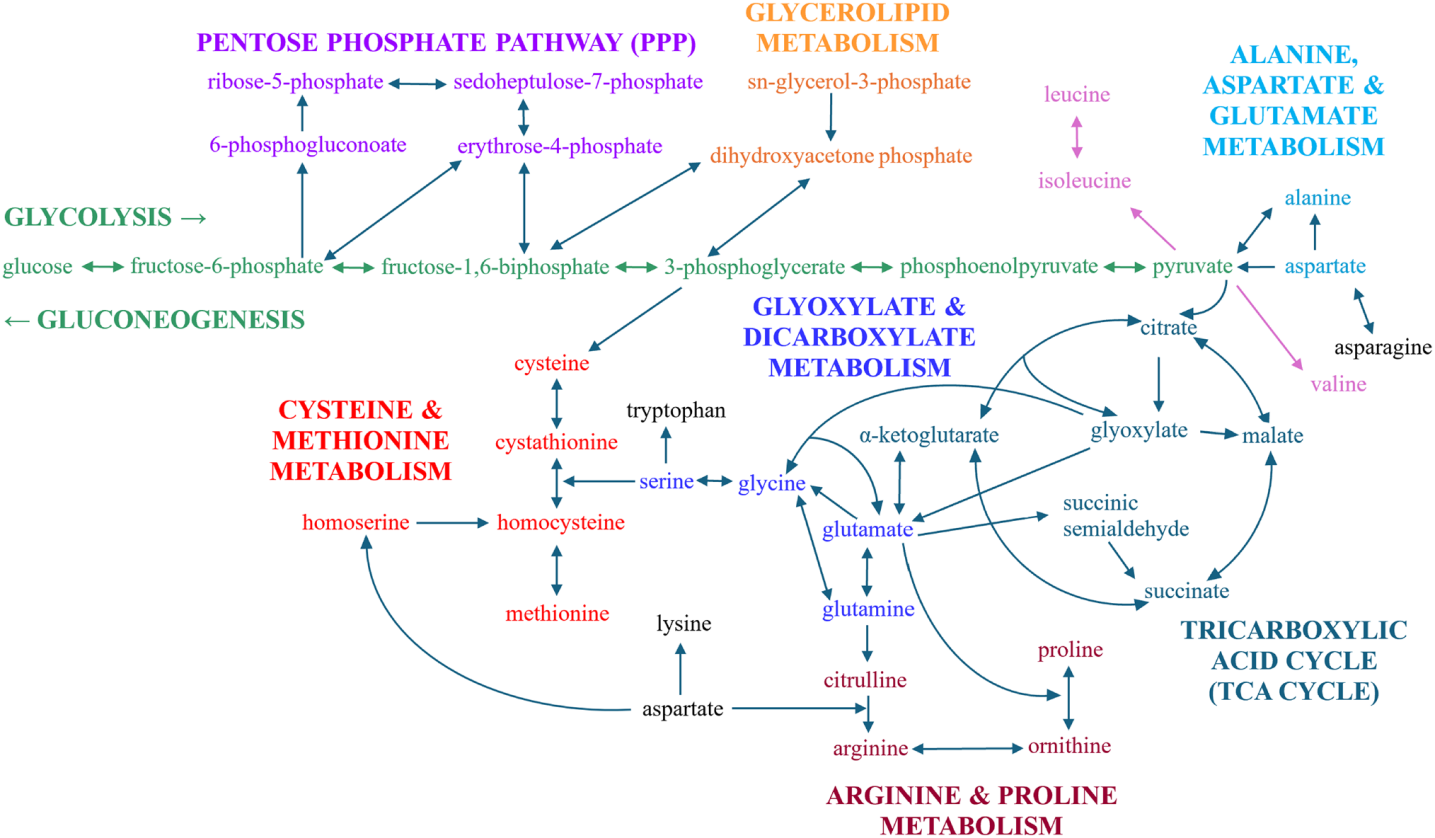
The MTP adhesin plays a role in modulation of glycolysis, TCA, PPP, amino acid synthesis and metabolism. Pathways affected by the deletion of MTP

**Fig. 5:**
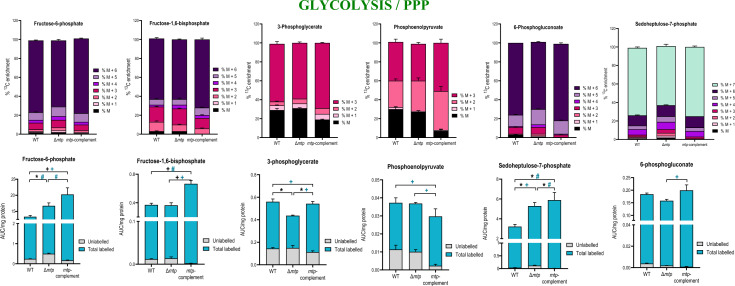
The MTP adhesin plays a role in modulation of glycolysis and PPP. The percent.^13^C_6_-glucose enrichment and carbon isotopologue distribution and total abundance (labelled and unlabelled) of compounds involved in glycolysis and PPP; fructose-6-phosphate showing a significantly lower % M + 5 species in the Δ*mtp* strain, fructose-1,6-bisphosphate, 3-phosphoglycerate depicting lower % M + 3 incorporation the Δ*mtp* strain, phosphoenolpyrvate, sedoheptulose-7-phosphate, and 6-phosphogluvonoate depicting significantly lower incorporation of labelled glucose in the Δ*mtp* strain in comparison to the wildtype V9124 (WT) and *mtp*-complement strains. Each column represents the mean of at least four replicates with the standard error of mean (SEM). This data is representative of one experiment, with the experiment being repeated a minimum of two times. A one-way ANOVA and Tukey multiple-comparison test were performed for the statistical analysis. The statistical tests were performed on the total abundance (black) and the total labelled abundance (blue) for each compound. *n* = four biologically independent samples. False discovery rate (FDR) adjusted *p*-values * *p* < 0.05, + *p* ≤ 0.01, # *p* ≤ 0.0001

**Fig. 6:**
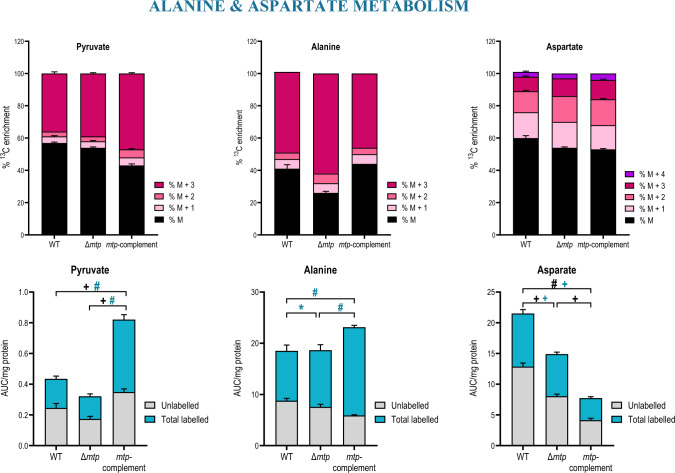
The MTP adhesin plays a role in modulation of alanine and aspartate metabolism. The percent.^13^C_6_-glucose enrichment and carbon isotopologue distribution and total abundance (labelled and unlabelled) of the compounds involved in alanine and aspartate metabolism; pyruvate and aspartate showing higher % M + 3 and % M + 4 species in the Δ*mtp* strain, and alanine depicting significantly lower incorporation of labelled glucose in the Δ*mtp* strain in comparison to the wildtype V9124 (WT) and *mtp*-complement strains. Each column represents the mean of at least four replicates with the standard error of mean (SEM). This data is representative of one experiment, with the experiment being repeated a minimum of two times. A one-way ANOVA and Tukey multiple-comparison test were performed for the statistical analysis. The statistical tests were performed on the total abundance (black) and the total labelled abundance (blue) for each compound. *n* = four biologically independent samples. False discovery rate (FDR) adjusted *p*-values * *p* < 0.05, + *p* ≤ 0.01, # *p* ≤ 0.0001

**Fig. 7:**
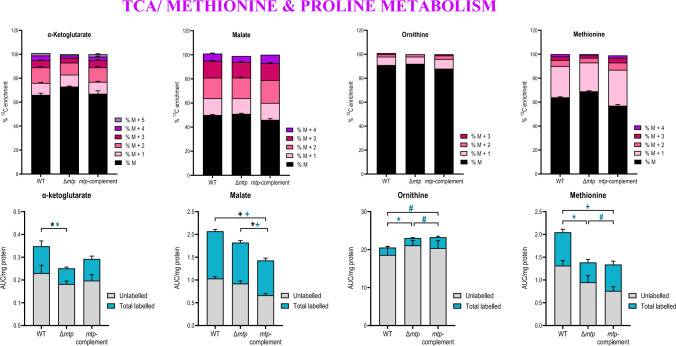
The MTP adhesin plays a role in modulation of TCA, methionine and proline metabolism. The percent ^13^C_6_-glucose enrichment and carbon isotopologue distribution and total abundance (labelled and unlabelled) in the following compounds: malate and α-ketoglutarate depicting significantly lower incorporation of labelled glucose in the Δ*mtp* strain, methionine, and ornithine, depicting significantly lower incorporation of labelled glucose in the Δ*mtp* strain in comparison to the wildtype V9124 (WT) and *mtp*-complement strains. Each column represents the mean of at least four replicates with the standard error of mean (SEM). This data is representative of one experiment, with the experiment being repeated a minimum of two times. A one-way ANOVA and Tukey multiple-comparison test were performed for the statistical analysis. The statistical tests were performed on the total abundance (black) and the total labelled abundance (blue) for each compound. *n* = four biologically independent samples. False discovery rate (FDR) adjusted *p*-values * *p* < 0.05, + *p* ≤ 0.01, # *p* ≤ 0.0001

Figure 4 illustrates the metabolic pathways that displayed alterations in metabolic fluxes amongst the strains, with the ^13^C_6_-glucose incorporation percentages per compound and the total abundance of each compound are depicted in Figs. 5, 6, 7.

A significant decrease in carbon incorporation in fructose-6-phosphate (*p* ≤ 0.0001) and no significant difference in fructose-1,6-bisphosphate (Fig. 5) was observed in the Δ*mtp* compared to the WT. The decreased flux in these compounds indicated a decreased flux in glycolysis in the absence of MTP. In addition, the total abundance of fructose-6-phosphate (*p* < 0.05) was significantly higher in the Δ*mtp* in comparison to the WT (Fig. 5), indicating an accumulation of the compound with decreased conversion into fructose-1,6-bisphosphate.

Fructose-1,6-bisphosphate is converted into 3-phosphoglycerate (Fig. 5) and dihydroxyacetone phosphate (Figure S2). The ^13^C-isotopomer analysis revealed a decrease in carbon incorporation in 3-phosphoglycerate (Fig. 5) and a slight decrease in ^13^C_6_-glucose incorporation in dihydroxyacetone phosphate (Figure S2). The total abundance analysis demonstrated a significantly lower abundance of 3-phosphoglycerate (Fig. 5) (*p* < 0.05) and higher abundance of dihydroxyacetone phosphate (Figure S3) (*p* ≤ 0.01) in the Δ*mtp* compared to the WT, suggesting increased flux in the conversion of fructose-1,6-bisphosphate into dihydroxyacetone phosphate.

During glycerolipid metabolism, dihdroxyacetone phosphate is converted to glycerol-3-phosphate by glycerol-3-phosphate dehydrogenenase (Baughn & Rhee, 2014; Rutter, 1964). An increase in glycerol-3-phosphate carbon incorporation (Figure S2) and significant increase in total abundance was observed in the Δ*mtp* compared to the WT. These data provide evidence of increased flux in glycerolipid metabolism and decreased flux in glycolysis intermediates, in the absence of MTP.

During glycolysis, 3-phosphoglycerate is converted to phosphoenolpyruvate which is then converted to pyruvate (Fig. 5 and 6) (Berg et al., 2007). The ^13^C-isotopomer analysis showed a slight increase in carbon incorporation in phosphoenolpyruvate and pyruvate in the Δ*mtp* compared to the WT, with no significant difference in total abundance observed amongst the strains (Fig. 5 and 6). Overall, the results reveal decreased flux in glycolysis and an enhanced flux into glycerolipid metabolism in the absence of MTP.

### The MTP adhesin is associated with modulation of the PPP in *Mtb*

The conversion of glucose-6-phosphate into 6-phosphoglucono-δ-lactone is the rate limiting step of the PPP. Phosphoglucono-δ-lactone is then converted to 6-phosphogluconoate which is converted to ribulose-5-phosphate and thereafter into ribose-5-phosphate to sedoheptulose-7-phosphate to erythose-4-phosphate (Baughn & Rhee, 2014; Griffin et al., 2011; Sassetti et al., 2003).

The ^13^C-isotopomer analysis revealed a lower level of carbon incorporation and lower abundance of 6-phosphogluconoate (Fig. 5) in the Δ*mtp* compared to the WT. The level of ^13^C_6_-glucose incorporation in ribose-5-phosphate was lower (Figure S2), with the total abundance (*p* < 0.05) showing a significant higher level in the Δ*mtp* compared to the WT (Figure S3). Sedoheptulose-7-phosphate displayed a significant decrease in carbon incorporation and a significantly decreased total abundance (Fig. 5) in the Δ*mtp* compared to the WT. In addition, a decreased carbon incorporation (Figure S3) and a significant increase in the total abundance of erythose-4-phosphate (*p* < 0.05) (Figure S3) was observed in the Δ*mtp* compared to the WT. Collectively, the Δ*mtp* displays a decreased flux through glycolysis and PPP, compared to the WT, potentially leading to decreased utilisation of pathway intermediates, and may result in the accumulation of erythose-4-phosphate observed.

### The absence of MTP dysregulates the TCA cycle

The ^13^C-isotopomer analysis revealed a lower level of carbon incorporation in citrate (Figure S2), malate (Fig. 7), glyoxylate and significantly decreased incorporation in α-ketoglutarate (*p* < 0.05) (Fig. 7) in the Δ*mtp* compared to the WT. A significantly decreased total labelled abundance of α-ketoglutarate (*p* < 0.05) and decreased total abundance of citrate (Figure S3) and malate (Fig. 7), was observed in Δ*mtp* compared to the WT. Overall, the reduced incorporation and total abundance indicates a reduced flux in the TCA cycle in the absence of MTP.

### MTP affects amino acid production

The significantly increased carbon incorporation in aspartate combined with the significant decrease in its total abundance (Fig. 6) suggest the increased flux through aspartate feeds into amino acid production in the Δ*mtp*. Arginine (*p* < 0.05) (SI Figure S3), lactate (*p* < 0.05) (SI Figure S3), and citrulline (*p* < 0.05) (SI Figure S3) displayed a significant increase in total abundance in the Δ*mtp* compared to the WT. In addition, the total abundance of asparagine (Figure S3), glutamate (Figure S3), and homocysteine (Figure S3) was significantly lower in the Δ*mtp* compared to the WT. Collectively, these results demonstrate the effect of MTP on amino acid regulation.

## Discussion

The present study demonstrated the modulatory role MTP plays in increasing bacterial respiration and decreasing carbon catabolism via glycolysis, thereby aiding the pathogen’s ability to maintain homeostasis and virulence. Furthermore, ^13^C-isotopomer metabolomic analysis revealed that MTP plays a regulatory role in CCM by maintaining the flux through glycolysis, TCA, glyoxylate and dicarboxylate metabolism, and PPP to maintain optimal metabolic functioning. The absence of MTP resulted in a decelerated CCM. Therefore, MTP plays a regulatory role in bioenergetic metabolism contributing to the virulence of *Mtb.*

### MTP plays a role in regulating basal OCR

Classified as an obligate aerobe, *Mtb* requires the use of its branched ETC for energy production through OXPHOS (Cook et al., 2014). With the use of XF analysis, OCR and ECAR were used as markers to measure OXPHOS and carbon catabolism (Lamprecht et al., 2016; Saini et al., 2016). The ∆*mtp* displayed a significantly higher basal OXPHOS in comparison to the WT (*p* ≤ 0.01) and *mtp*-complement (*p* ≤ 0.01). These findings corroborate the findings from a previous RNA sequencing study which identified the significant up-regulation of genes involved in complex IV and V (ATP synthase) of the OXPHOS pathway in the ∆*mtp* compared to the WT and *mtp*-complement, suggesting that the absence of MTP increases basal OXPHOS in the pathogen (Naidoo et al., 2025). Collectively, these data suggest that the pathogen attempts to increase ATP synthesis via OXPHOS due to decrebliased proton motive gradient (Bald and Koul, 2010), which was hypothesized to be perturbed by the absence of the surface located MTP adhesin (Naidoo, 2021). The increased basal OXPHOS measurements in the present study support the previous hypothesis by Naidoo, 2021, that the deletion of MTP alters the proton gradient by perturbing the ETC which leads to decreased ATP synthesis by this pathway and subsequently increasing ATP generation via complex V of the OXPHOS pathway.

The change in OCR and ECAR was calculated using baseline (measurement 4) and response to the ETC targeting drug BDQ (measurement 8; Figs. 2–3). Consequently, the ∆*mtp* displayed a significantly lower change in OCR (Fig. 2) compared to the WT (*p* ≤ 0.01) and *mtp*-complement (*p* ≤ 0.01). Since BDQ is an ETC targeting drug, the OCR measurements were expected to decrease after its addition. However, the OCR and ECAR increased in all strains compared to the respective controls (Figs. 2–3). A previous bioenergetics study investigating the effect of BDQ on *Mtb* proposed the increase in OCR observed, was a result of the pathogen shifting from OXPHOS to other energy generating pathways to maintain homeostasis (Mackenzie et al., 2020). Since BDQ inhibits ATP synthase activity, ATP levels decrease from this pathway. These decreased ATP levels result in increased activity of energy generating pathways to maintain homeostasis, resulting in an increase in OCR parameters (Lamprecht et al., 2016). The significant decrease in the change in OCR observed in the ∆*mtp* in response to the inhibition of OXPHOS, indicates a decrease in bacterial respiration in comparison to the WT and *mtp*-complement. This suggests that in the absence of MTP, the pathogen’s ability to increase OXPHOS, by increasing activity of energy generating pathways is potentially hindered. The increase in OXPHOS observed in the WT in the present study and previous studies, may be a compensatory mechanism employed by the pathogen due to the decrease in ATP levels to maintain homeostasis. Hence, in the absence of MTP, the mutant potentially displays a greater dependence on OXPHOS for energy production than the WT and *mtp*-complement. In addition, the lack of MTP may increase the pathogen’s affinity to BDQ and may increase pathogen killing by BDQ.

### MTP plays a role in maintaining energy homeostasis in *Mtb*

The basal ECAR measurements demonstrated no significant difference amongst the strains (Fig. 3). However, the ∆*mtp* displayed a significantly increased change in ECAR in comparison to the WT (*p* < 0.05). This suggests a significant increase in carbon catabolism via glycolysis and the TCA cycle in the *∆mtp* in comparison to the WT. Collectively, these data reveal that inhibition of OXPHOS hinders the ability of the mutant to optimally increase OCR following BDQ addition in an effort to maintain energy homeostasis and instead increases carbon catabolism as an alternative energy generating pathway. This suggests that the presence of MTP functions in part to maintain energy homeostasis in the pathogen.

### MTP plays a role in regulation of central carbon metabolism

The carbon metabolism of *Mtb* is a significant determinant of its ability to replicate and survive within a host cell (Ehrt et al., 2018; Marrero et al., 2010; Quinonez et al., 2022). Defining the perturbations to the metabolic pathways of the *∆mtp* is essential to understand its pathogenicity and to provide a guide for the development of new therapeutics. The maintenance of membrane function has been strongly implicated in the functioning of metabolic enzymes required for the persistence and survival of *Mtb *in vitro (Ehrt et al., 2018; Hartman et al., 2017; Machová et al., 2014; Marrero et al., 2010; Wayne & Hayes, 1996). Hence, the perturbation to membrane function caused by the deletion of MTP resulted in significant alterations to metabolic functioning of the *Mtb* deletion mutant. The metabolic profile of *∆mtp*, determined by ^13^C-isotopomer analysis, revealed a significant decrease in flux through glycolysis, PPP, TCA cycle, and an enhanced flux through glycerolipid metabolism (Figs. 4–7).

#### MTP enhances flux through glycolysis and decreases flux through glycerolipid metabolism

The conversion of fructose-6-phosphate and ATP into fructose-1,6-bisphosphate and ADP is catalysed by phosphofructokinase. This first irreversible reaction is the rate-limiting step of glycolysis (Benrahmoune et al., 2000). The decreased flux in both compounds and increased abundance of fructose-6-phosphate (Fig. 5) further supports the evidence of an accumulation of the compound with decreased incorporation into fructose-1,6-bisphosphate. The decrease in the total abundance of 3-phosphoglycerate and an increase in the total abundance of dihydroxyacetone, in conjunction with the decreased ^13^C_6_-glucose incorporation in both compounds, points towards the mutant preferentially converting fructose-1,6-bisphosphate into dihydroxyacetone phosphate instead of the glycolysis intermediate. During glycolysis, 3-phosphoglycerate is converted into phosphoenolpyruvate which is converted into pyruvate (Fig. 5 and 6) (Berg et al., 2007). An increase in the total abundance and ^13^C_6_-glucose incorporation of glycerol-3-phosphate in the *∆mtp* (Figure S2) supports the previous assumption that the mutant preferentially converts fructose-1,6-bisphosphate into dihydroxyacetone phosphate toward glycerolipid metabolism instead of glycolysis intermediates. Overall, the results revealed an enhanced flux into glycerolipid metabolism possibly resulting in the decreased flux through glycolysis in the absence of MTP. Due to their categorization as membrane lipids (Holzheimer et al., 2021; Hunter et al., 1986), and stored energy sources in *Mtb* (Pal et al., 2017; Sieniawska et al., 2020), the preference toward glycerolipid metabolism in the *∆mtp* may be attributed to the perturbation in the cell wall, potentially causing the pathogen to favour glycerolipid metabolism in an effort to maintain cell wall functioning.

#### MTP plays a role in regulating the PPP

Lower glycolysis is fuelled by reducing equivalents, nucleotides, and triose intermediates produced by the PPP (Baughn & Rhee, 2014; Griffin et al., 2011; Sassetti et al., 2003). The PPP aids in carbon homeostasis (Baughn & Rhee, 2014; Griffin et al., 2011; Sassetti et al., 2003). The metabolic profiling findings in the present study suggest higher ^13^C_6_-glucose incorporation and a decrease in the total abundance of 6-phosphogluconoate, and a lower ^13^C_6_-glucose incorporation with an increase in the total abundance of sedoheptulose-7-phosphate in the *∆mtp*. In addition, the lower ^13^C_6_-glucose incorporation and increased total abundance of ribose-5-phosphate and erythose-4-phosphate in the *∆mtp*, suggests a decreased flux though the PPP potentially leading to the decreased utilisation of pathway intermediates resulting in their accumulation in the absence of MTP. Furthermore, the decreased flux through the PPP potentially resulted in the decrease in flux observed through lower glycolysis in the *∆mtp*. The alterations to the PPP potentially result in decreased reducing molecules for subsequent metabolic processes such as glycolysis, amino acid production, and the dysregulation of carbon homeostasis (Stincone et al., 2015).

#### The absence of MTP is associated with a decreased flux through the TCA cycle and glyoxylate shunt

The epicentre of CCM, the TCA cycle, is responsible for the generation of substrates required for OXPHOS, energy production, and biosynthesis of essential for amino acids and lipids (Cole et al., 1998; Xu & Borah, 2022). Previous in vitro metabolomic studies demonstrated the discontinuous carbon flow through the TCA cycle, between the TCA cycle intermediate, α-ketoglutarate, and succinate in *Mtb* cultures (de Carvalho et al., 2010; Rhee et al., 2011; Tian et al., 2005; Xu & Borah, 2022). The conversion of isocitrate to α-ketoglutarate is also regarded as the rate-limiting step of the TCA cycle (Kondrashov et al., 2006; Quartararo et al., 2013).

The variant of the TCA cycle proposed was the glyoxylate shunt, which bypasses the carbon oxidation via the oxidative branch of the TCA cycle (Borah et al., 2021; McKinney et al., 2000; Murima et al., 2016; Serafini et al., 2019). Several studies have demonstrated the importance of the glyoxylate shunt in *Mtb* growth on cholesterol, fatty acids, and acetate (Borah et al., 2021; McKinney et al., 2000; Murima et al., 2016; Serafini et al., 2019). Malate synthase and isocitrate lyase are two enzymes involved in the glyoxylate shunt. These enzymes facilitate the preservation and replenishment of carbon from the TCA cycle intermediates by synthesising glyoxylate and succinate from the precursor isocitrate (Cole et al., 1998; Marrero et al., 2010; Muñoz‐Elías et al., 2006; Puckett et al., 2017). The glyoxylate shunt aids in *Mtb* survival under various conditions which include antibiotic stress, hypoxia, and oxidative stress (Ahn et al., 2016; Eoh & Rhee, 2013, 2014). *Mtb* utilises β-oxidation to degrade fatty acids into acetyl-coenzyme A and thereafter, acetate, which enters the TCA and is oxidised to generate substrates required for ATP production (Murima et al., 2016). Moreover, *Mtb* is able to facilitate the oxidation of lactate to pyruvate, compounds required in the TCA and methylcitrate cycle, glyoxylate shunt, and valine degradation (McKinney et al., 2000, Muñoz‐Elías et al., 2006, Marrero et al., 2010, Eoh & Rhee, 2013, Eoh & Rhee, 2014, Ahn et al., 2016, Serafini et al., 2019, Borah et al., 2021).

In the present study, metabolic profiling revealed that the absence of MTP dysregulates the TCA cycle. A decreased total abundance and ^13^C_6_-glucose incorporation in citrate (SI Figure S2), malate (Fig. 7), and α-ketoglutarate (Fig. 7) was observed in the *∆mtp*. In addition, a decreased carbon incorporation was observed in glyoxylate (SI Figure S2) in the *∆mtp*. Collectively, the metabolic profiling data revealed a decreased flux in the TCA cycle and glyoxylate shunt in the *∆mtp* suggesting that MTP may play a role in regulating optimal functioning of these crucial pathways. Furthermore, the decreased flux in the TCA cycle and glyoxylate shunt may result in decreased ATP production via these metabolic pathways, thereby potentially increasing ATP production via ATP synthase in the OXPHOS pathway in an effort to maintain energy homeostasis in the absence of MTP.

#### MTP plays a role in regulation of amino acids involved in survival and replication of *Mtb*

In addition to carbon, nitrogen is an alternative building block for biomass such as amino acids, lipids, nucleic acids, and proteins (Xu & Borah, 2022). In vitro studies revealed that *Mtb* preferentially utilises amino acids such as asparagine, aspartate, glutamine and glutamate over other inorganic sources of nitrogen (Agapova et al., 2019). The significantly increased ^13^C_6_-glucose incorporation in aspartate (Fig. 6) and alanine (Fig. 6) coupled with the decreased total abundance of aspartate, suggests that the *∆mtp* increases flux through aspartate subsequently feeding into amino acid production. In addition, arginine (*p* < 0.05) (Figure S3), lactate (*p* < 0.05) (Figure S3), ornithine (Fig. 7), and citrulline (*p* < 0.05) (Figure S3) displayed a significantly increased total abundance and decreased ^13^C_6_-glucose incorporation in the *∆mtp*. Asparagine, glutamate, and homocysteine showed a decrease in both the total abundance and ^13^C_6_-glucose incorporation in the *∆mtp*. This suggests an overall decreased utilisation of these compounds in subsequent metabolic reactions, potentially hindering the growth of the pathogen.

The aspartate catabolic pathway plays a significant role in the synthesis of essential amino acids such as lysine, isoleucine, methionine, and threonine (Berney et al., 2015; Gordhan et al., 2002). Moreover, the aspartate catabolic pathway is involved in the synthesis of alanine which serves as a precursor required for pantothenic acid synthesis in *Mtb* (Berney et al., 2015). Thus, the increased flux into aspartate coupled with the increased flux through alanine and decreased flux in isoleucine (Figure S2), lysine, methionine, and threonine (Figure S2) point toward the aspartate catabolic pathway feeding into alanine production in the absence of MTP.

The arginine biosynthetic enzyme is essential for *Mtb* and its homologous gene is absent in humans (Yelamanchi & Surolia, 2021). Arginine also serves as a carbon source for proline and ornithine synthesis (Hampel et al., 2015). Ornithine is then catalysed into citrulline (Gordhan et al., 2002). In the present study, the increase in total abundance of arginine coupled with the decreased flux observed in the *∆mtp*, resulted in the decreased synthesis of ornithine and citrulline which is substantiated by the decreased flux in these compounds. The decreased flux through these compounds in combination with the increase in the total abundance point toward a decreased utilisation in subsequent metabolic processes. Since these compounds play a role in bacterial survival and replication (Gordhan et al., 2002; Hampel et al., 2015; Yelamanchi & Surolia, 2021), the decreased utilisation hinders growth of *Mtb.*

Previous reports have identified asparagine as the sole nitrogen donor to glutamate and glutamine (Gouzy et al., 2014). Hence, the decreased flux in asparagine resulted in the decreased synthesis observed in the *∆mtp*, in both glutamate and glutamine in the present study (Figure S2 and S3). In vivo and in vitro studies on glutamate dehydrogenase, responsible for the conversion of glutamate to ammonia and 2-oxoglutarate, identified its essentiality in *Mtb* survival and resistance to stress responses (Gallant et al., 2016; Gordon et al., 1980). Therefore, the decreased flux and abundance of these amino acids may hinder the growth of *Mtb* in the absence of MTP. This study highlighted the significance of MTP in modulation of OXPHOS and CCM in *Mtb*, and reported a significant increase in OXPHOS coupled with a decreased flux through glycolysis, the TCA cycle, glyoxylate shunt, and pentose phosphate pathway in the *∆mtp*. These results validate previous studies which suggest that MTP plays a dual role in adhesion and regulation of *Mtb* CCM (Ashokcoomar et al., 2021, 2025; Naidoo et al., 2025) Despite these strengths, this study is limited to a bacterial model, which does not replicate the complex dynamics of host–pathogen interactions, therefore limiting the translatability to human infection. However, in mitigation of this, a recent study by our group (Ashokcoomar et al., 2025) elucidated the effect of MTP on the bioenergetic and metabolomic profiles of THP-1 macrophages. The *∆mtp* infected THP-1 macrophages and uninfected THP-1 macrophages exhibited higher oxygen consumption rates (OCR), in comparison with the WT and *mtp*-complement infected THP-1 macrophages (Ashokcoomar et al., 2025). In addition, a decrease in the total abundance of metabolites was observed in the uninfected THP-1 macrophages and the ∆*mtp* infected THP-1 macrophages, suggesting that the absence of MTP enables swift clearance of the intracellular *Mtb*. This suggests that the presence of MTP facilitates the survival of the pathogen during early stages until infection is established (Ashokcoomar et al., 2025). These findings support the current data, which suggests that MTP plays a role in the regulation of bioenergetics and metabolism pathways.

A further limitation is that due to financial constraints, and the large volume of data generated by a study of this nature, only one clinical strain was used as the WT, thus limiting extrapolation to all clinical strains. Future studies would require the use of different strain families in which *mtp* has been knocked out.

In conclusion, the absence of the surface located mycobacterial adhesin, MTP, is significantly associated with: 1) perturbations to the pathogen’s membrane function, potentially resulting in dysregulation of several metabolic pathways; 2) potentially greater ATP demand leading to the increase in OXPHOS in an effort to meet the energy demand; and 3) decreased flux through amino acids involved in the survival and replication of *Mtb*, thereby potentially hindering the growth and pathogenicity of the pathogen. Collectively, this study demonstrated the significant role MTP plays in regulation of OXPHOS and CCM*.* Future studies should focus on the host–pathogen interaction to better understand the role MTP plays in regulation of *Mtb* metabolism in vitro as well as in vivo. This study provides new knowledge on the role of MTP in survival and replication of *Mtb*, and explains the metabolic mechanisms involved in the reduced *Mtb ∆mtp* growth observed in previous adhesion and invasion studies (Govender et al., 2018; Moodley, 2018; Ramsugit et al., 2013, 2016). Thus, the findings further substantiate MTP as an eminent biomarker which should be targeted for the development of anti-TB drugs and vaccines.

## Supplementary Information

Below is the link to the electronic supplementary material.

Supplementary file1 (DOCX 919 KB)

## Data Availability

Data is provided within the manuscript or supplementary information files.
