# Supplementary material for: Mycobacterium tuberculosis curli pili facilitates pathogenicity by modulating central carbon metabolism
Source: Metabolomics. 2025 Aug 12;21(5):118. doi: 10.1007/s11306-025-02320-5 (PMC12343700; doi:10.1007/s11306-025-02320-5)
Supplement: Supplementary file 1 — Supplementary file1 (DOCX 919 KB) [file 11306_2025_2320_MOESM1_ESM.docx]

**Supplementary Information (SI)**

**Part A: Extracellular flux (XF) assay**

The normalized data was analyzed on the software mentioned in the methodology section. The statistical analysis was performed on the normalized data. A one-way ANOVA with Tukey’s multiple-comparison was performed on the original baseline measurements for the OCR (Table S1) and ECAR (Table S2).

Table S1: One-way ANOVA significance results with Tukey’s multiple-comparison test to determine statistical significance amongst strains for their baseline OCR measurements

| Tukey's multiple comparisons test | Significant? | Adjusted *p* Value |
| --- | --- | --- |
| ANOVA | Yes | 0,0004 |
| Wildtype vs. *∆mtp* | Yes | 0,0005 |
| Wildtype vs. *mtp-*complement | No | 0,4873 |
| *∆mtp* vs. *mtp-*complement | Yes | 0,0035 |

Table S2: One-way ANOVA significance results with Tukey’s multiple-comparison test to determine statistical significance amongst strains for their baseline ECAR measurements

| Tukey's multiple comparisons test | Significant? | Adjusted *p* Value |
| --- | --- | --- |
| ANOVA | No | 0,2549 |
| Wildtype vs. *∆mtp* | No | 0,6164 |
| Wildtype vs. *mtp-*complement | No | 0,7917 |
| *∆mtp* vs. *mtp-*complement | No | 0,2275 |

Following the baseline measurement statistical analysis, a one-way ANOVA with Tukey’s multiple-comparison test was performed on the calculated change in OCR (Table S3) and change in ECAR (Table S4).

Table S3: One-way ANOVA significance results with Tukey’s multiple-comparison test to determine statistical significance amongst *M. tuberculosis* strains for the change in OCR

| Tukey's multiple comparisons test | Significant? | Adjusted *p* Value |
| --- | --- | --- |
| ANOVA | Yes | 0,0019 |
| Wildtype vs. *∆mtp* | Yes | 0,0058 |
| Wildtype vs. *mtp-*complement | No | 0,9981 |
| *∆mtp* vs. *mtp-*complement | Yes | 0,0036 |

Table S4: One-way ANOVA significance results with Tukey’s multiple-comparison test to determine statistical significance amongst strains for the change in ECAR

| Tukey's multiple comparisons test | Significant? | Adjusted *p* Value |
| --- | --- | --- |
| ANOVA | Yes | 0,0006 |
| Wildtype vs. *∆mtp* | Yes | 0,0297 |
| Wildtype vs. *mtp-*complement | Yes | 0,0004 |
| *∆mtp* vs. *mtp-*complement | No | 0,1057 |

**Part B: Metabolomics**

Normalization

The data was initially normalized using the BCA protein concentrations and internal standards (methodology section). The protein concentrations were calculated using the BCA absorbance according to the kit (methodology), using the mentioned standards.

Table S5: The absorbance (540_nm_) and calculated protein concentrations (ug/mL) of the wild-type, *∆mtp*, and *mtp-*complement samples

| **Sample** | **Absorbance (540_nm_)** | **Protein concentrations (ug/mL)** |
| --- | --- | --- |
| Wildtype 1 | 0.426 | 465.11 |
| Wildtype 2 | 0.484 | 529.56 |
| Wildtype 3 | 0.538 | 589.56 |
| Wildtype 4 | 0.528 | 578.44 |
| Wildtype 5 | 0.521 | 570.67 |
| *∆mtp* 1 | 0.507 | 555.11 |
| *∆mtp* 2 | 0.608 | 667.33 |
| *∆mtp* 3 | 0.496 | 542.89 |
| *∆mtp* 4 | 0.599 | 657.33 |
| *∆mtp* 5 | 0.579 | 635.11 |
| *mtp-*complement 1 | 0.479 | 524.00 |
| *mtp-*complement 2 | 0.409 | 446.22 |
| *mtp-*complement 3 | 0.357 | 388.44 |
| *mtp-*complement 4 | 0.426 | 465.11 |
| *mtp-*complement 5 | 0.383 | 417.33 |

Figure S1: Line graph representing the protein concentrations (ug/mL) at the specific absorbance (540_nm_)

Statistical analysis

A one-way ANOVA with Tukey’s multiple-comparisons was performed on the total labelled and total abundance data. The results are show in in Tables 7-8, respectively

| Table S6: One-way ANOVA with Tukey’s multiple-comparison statistical test for total abundance | | | | |
| --- | --- | --- | --- | --- |
|  | Adjusted p-value | | | |
| Compound | ANOVA | WT vs *∆mtp* | WT vs *mtp-*complement | *mtp* vs *mtp-*complement |
| sn-glycerol-3-phosphate | <0,0001 | 0.00370 | 0.01410 | <0,0001 |
| 3-phosphoglycerate | 0.02170 | 0.03820 | 0.99550 | 0.03320 |
| Phosphoenolpyruvate | 0.69200 | >0,9999 | 0.73340 | 0.73920 |
| Pyruvate | 0.00010 | 0.22900 | 0.00090 | 0.00010 |
| lactate | 0.02640 | 0.02130 | 0.23620 | 0.30230 |
| Citrate | 0.00680 | 0.22400 | 0.08340 | 0.00540 |
| Succinate | 0.00300 | 0.46500 | 0.01740 | 0.00290 |
| Fumarate | 0.00260 | 0.26120 | 0.00220 | 0.02570 |
| Malate | 0.00200 | 0.16780 | 0.00160 | 0.02920 |
| Glyoxylate | 0.57700 | 0.99570 | 0.66010 | 0.60800 |
| Methylcitrate | <0,0001 | <0,0001 | <0,0001 | 0.00670 |
| alpha-Ketoglutarate | 0.04300 | 0.03570 | 0.24970 | 0.43700 |
| Itaconic acid | 0.32940 | 0.84160 | 0.59390 | 0.30770 |
| Citramalate | 0.09320 | 0.70640 | 0.27810 | 0.08470 |
| Methylsuccinate | <0,0001 | <0,0001 | <0,0001 | 0.97070 |
| Dihydroxyacetonephosphate  (DHAP) | 0.00180 | 0.00160 | 0.01750 | 0.26700 |
| D-Erythose-4-phosphate | 0.00480 | 0.03240 | 0.00430 | 0.40490 |
| Ribose-5-phosphate | 0.01070 | 0.02060 | 0.01700 | 0.99180 |
| Fructose-6-phosphate | 0.00740 | 0.04210 | 0.00680 | 0.48350 |
| Fructose-1,6-biphosphate | 0.00060 | 0.98060 | 0.00140 | 0.00110 |
| Sedoheptulose-7-phosphate | 0.01220 | 0.04740 | 0.01230 | 0.66900 |
| Glyceraldehyde-DHA | 0.00210 | 0.83040 | 0.00650 | 0.00280 |
| Erythrose | 0.00300 | 0.98490 | 0.00650 | 0.00510 |
| Ribose-ribulose | 0.00150 | 0.77040 | 0.00510 | 0.00190 |
| Hexose | 0.00510 | 0.99970 | 0.00950 | 0.00920 |
| Sedoheptulose | <0,0001 | <0,0001 | 0.00390 | 0.01440 |
| Trehalose | 0.00030 | 0.65910 | 0.00040 | 0.00130 |
| Succinic semialdehyde | 0.00010 | 0.99970 | 0.00030 | 0.00030 |
| 6-phosphogluconate | 0.14630 | 0.14300 | 0.87280 | 0.29150 |
| Alanine | 0.05170 | 0.99670 | 0.07350 | 0.08280 |
| Arginine | 0.00320 | 0.04330 | 0.19520 | 0.00260 |
| Asparagine | 0.00040 | 0.01590 | 0.03350 | 0.00030 |
| Aspartate | <0,0001 | 0.00060 | <0,0001 | 0.00030 |
| Cysteine | 0.12690 | 0.99670 | 0.18260 | 0.16310 |
| Glycine | 0.01300 | 0.77290 | 0.04170 | 0.01430 |
| Glutamate | 0.01670 | 0.01340 | 0.15200 | 0.30400 |
| Glutamine | 0.01290 | 0.39380 | 0.09030 | 0.01080 |
| Isoleucine | 0.00200 | 0.92150 | 0.00300 | 0.00510 |
| Leucine | 0.29220 | 0.27540 | 0.53280 | 0.85330 |
| Histidine | 0.04470 | 0.41910 | 0.27000 | 0.03690 |
| Lysine | 0.45340 | 0.90320 | 0.43240 | 0.67750 |
| Methionine | 0.04130 | 0.07300 | 0.05600 | 0.98420 |
| Phenylalanine | 0.01100 | 0.32030 | 0.09560 | 0.00900 |
| Threonine | 0.05290 | 0.08050 | 0.07830 | 0.99980 |
| Homoserine | 0.07640 | 0.08230 | 0.16160 | 0.89750 |
| Proline | 0.25700 | 0.73080 | 0.59070 | 0.23160 |
| Serine | 0.00450 | 0.10660 | 0.00350 | 0.11330 |
| Tryptophan | 0.00500 | 0.14610 | 0.09210 | 0.00390 |
| Valine | 0.01600 | 0.07290 | 0.58070 | 0.01490 |
| Tyrosine | 0.09580 | 0.09230 | 0.23880 | 0.80070 |
| Homocysteine | <0,0001 | <0,0001 | <0,0001 | 0.04120 |
| Cystathionine | 0.20150 | 0.21880 | 0.96190 | 0.31460 |
| Ornithine | 0.61170 | 0.68700 | 0.64370 | 0.99700 |
| Citrulline | 0.01250 | 0.03280 | 0.01550 | 0.87910 |
| Ergothioneine | 0.00580 | 0.00550 | 0.52440 | 0.03010 |
| Glutathione | 0.31510 | 0.31930 | 0.94440 | 0.47390 |

| Table S7: One-way ANOVA with Tukey’s multiple-comparison statistical test for percentage of total labelled abundance | | | | |
| --- | --- | --- | --- | --- |
|  | Adjusted *p*-value | | | |
| Compound | ANOVA | WT vs *∆mtp* | WT vs *mtp-*complement | *∆mtp* vs *mtp-*complement |
| sn-glycerol-3-phosphate | <0,0001 | 0.5984 | <0,0001 | <0,0001 |
| 3-phosphoglycerate | 0.0002 | 0.7266 | 0.0008 | 0.0003 |
| Phosphoenolpyruvate | <0,0001 | 0.6823 | 0.0001 | 0.0004 |
| Pyruvate | <0,0001 | 0.1385 | <0,0001 | <0,0001 |
| lactate | 0.7088 | >0,9999 | 0.5773 | 0.5769 |
| Citrate | 0.7718 | 0.8115 | 0.8134 | 0.4597 |
| Succinate | <0,0001 | 0.5130 | 0.0001 | <0,0001 |
| Fumarate | 0.9921 | 0.9914 | 0.9971 | 0.9985 |
| Malate | 0.0025 | 0.6477 | 0.0106 | 0.0028 |
| Glyoxylate | 0.3394 | 0.3193 | 0.8577 | 0.5915 |
| Methylcitrate | 0.0005 | 0.9999 | 0.0010 | 0.0010 |
| alpha-Ketoglutarate | 0.0407 | 0.0397 | 0.7410 | 0.1265 |
| Itaconic acid | 0.7856 | 0.9993 | 0.8095 | 0.8282 |
| Citramalate | 0.0036 | 0.1903 | 0.0028 | 0.0493 |
| Methylsuccinate | 0.0059 | 0.8962 | 0.0079 | 0.0154 |
| Dihydroxyacetonephosphate  (DHAP) | 0.0011 | 0.0989 | 0.0237 | 0.0008 |
| D-Erythose-4-phosphate | 0.0340 | 0.4460 | 0.1987 | 0.0284 |
| Ribose-5-phosphate | 0.0505 | 0.9875 | 0.0861 | 0.0681 |
| Fructose-6-phosphate | <0,0001 | <0,0001 | <0,0001 | <0,0001 |
| Fructose-1,6-biphosphate | 0.0001 | 0.1720 | 0.0001 | 0.0011 |
| Sedoheptulose-7-phosphate | <0,0001 | 0.0012 | <0,0001 | <0,0001 |
| Glyceraldehyde-DHA | 0.5171 | 0.9154 | 0.4971 | 0.7294 |
| Erythrose | 0.0010 | 0.0034 | 0.7879 | 0.0014 |
| Ribose-ribulose | 0.1561 | 0.5830 | 0.5249 | 0.1356 |
| Hexose | 0.1712 | 0.5229 | 0.6242 | 0.4199 |
| Sedoheptulose | <0,0001 | 0.0006 | <0,0001 | 0.0317 |
| Trehalose | <0,0001 | 0.0759 | <0,0001 | <0,0001 |
| Succinic semialdehyde | 0.0665 | 0.9541 | 0.0794 | 0.1243 |
| 6-phosphogluconate | 0.0033 | 0.0520 | 0.1653 | 0.0026 |
| Alanine | <0,0001 | 0.0166 | <0,0001 | <0,0001 |
| Arginine | <0,0001 | 0.1235 | 0.0004 | <0,0001 |
| Asparagine | <0,0001 | <0,0001 | 0.0222 | 0.0003 |
| Aspartate | 0.0023 | 0.0063 | 0.0034 | 0.8998 |
| Cysteine | <0,0001 | <0,0001 | <0,0001 | <0,0001 |
| Glycine | <0,0001 | 0.7363 | <0,0001 | <0,0001 |
| Glutamate | 0.0297 | 0.2936 | 0.0240 | 0.2707 |
| Glutamine | 0.0002 | 0.0040 | 0.0529 | 0.0001 |
| Isoleucine | <0,0001 | 0.2466 | <0,0001 | <0,0001 |
| Leucine | <0,0001 | 0.8505 | <0,0001 | <0,0001 |
| Histidine | 0.0034 | 0.5148 | 0.0181 | 0.0034 |
| Lysine | <0,0001 | 0.6445 | <0,0001 | 0.0001 |
| Methionine | <0,0001 | 0.0118 | 0.0004 | <0,0001 |
| Phenylalanine | <0,0001 | 0.8084 | <0,0001 | <0,0001 |
| Threonine | <0,0001 | 0.8338 | <0,0001 | <0,0001 |
| Homoserine | <0,0001 | 0.8503 | <0,0001 | <0,0001 |
| Proline | <0,0001 | 0.6736 | <0,0001 | <0,0001 |
| Serine | <0,0001 | 0.2021 | <0,0001 | 0.0001 |
| Tryptophan | <0,0001 | 0.0414 | <0,0001 | 0.0005 |
| Valine | 0.0103 | 0.0850 | 0.0085 | 0.3374 |
| Tyrosine | <0,0001 | 0.7032 | <0,0001 | <0,0001 |
| Ornithine | <0,0001 | 0.0089 | <0,0001 | <0,0001 |
| Citrulline | <0,0001 | 0.0004 | <0,0001 | <0,0001 |
| Ergothioneine | 0.9624 | 0.9654 | 0.9995 | 0.9727 |
| GSH | 0.5779 | 0.8626 | 0.8452 | 0.5491 |
| sn-glycerol-3-phosphate | <0,0001 | 0.5984 | <0,0001 | <0,0001 |
| 3-phosphoglycerate | 0.0002 | 0.7266 | 0.0008 | 0.0003 |

**Figure S2. The percent ^13^C enrichment and carbon isotopologue distribution of the 56 compounds analysed.** This data is representative of one experiment, with the experiment being repeated a minimum of two times. Each column represents the mean of at least four replicates with the standard error of mean (SEM).

**Figure S3. The total abundance of the 56 compounds analysed.** This data is representative of one experiment, with the experiment being repeated a minimum of two times. Each column represents the mean of atleast four replicates with the standard error of mean (SEM). A one-way ANOVA with Tukeys’s multiple-comparison test was performed for the statistical analysis. The statistical tests were performed on the total abundance (black) and the total labelled abundance (blue) for each compound. *n* = four biologically independent samples. False discovery (FDR) adjusted *p* value **p* < 0.05, + *p* ≤ 0.01, # *p* ≤ 0.0001.
